# The value of dual-energy CT virtual monoenergetic imaging and metal-artifacts reduction system after metal plate correction of congenital funnel chest in child: a single center retrospective study

**DOI:** 10.3389/fped.2025.1430160

**Published:** 2025-10-23

**Authors:** Chenglong Li, Chao Xu, Liya Lu, Lina Dong, Peng Du

**Affiliations:** ^1^ Department of Radiology, The Affiliated Xuzhou Children's Hospital of Xuzhou Medical University, Xuzhou, China; ^2^ Department of Radiology, The Second Affiliated Hospital of Xuzhou Medical University, Xuzhou, China

**Keywords:** computed tomography, dual-energy CT, virtual monoenergetic imaging, metal artifact reduction, congenital funnel chest, artifact, child

## Abstract

**Objective:**

This study aimed to evaluate the utility of virtual monoenergetic (MONO) imaging and a Metal-Artifact Reduction System (MARs) algorithm for enhancing image quality in dual-energy computed tomography (DECT) of children with congenital funnel chest (CFC) following minimally invasive correction with stainless steel plate implantation.

**Materials and methods:**

We retrospectively analyzed children with CFC who underwent thoracoscopic correction at our institution (January 2022 to August 2023). Their postoperative non-contrast chest CT scans were evaluated. Objective metrics (CT numbers, standard deviation-SD) were measured on five image series (120 kVp-like, 50 keV, 50 keV + MARs, 110 keV, 110 keV + MARs), and the absolute CT difference (|ΔCT|) and artifact index (AI) were calculated. Subjective image quality was scored by two independent radiologists. Given the non-normal distribution of data (Shapiro–Wilk test, *P* < 0.001), non-parametric tests (Friedman test with Wilcoxon *post hoc* analysis) were used for comparisons.

**Results:**

A total of 114 children were enrolled. Objective metrics (SD, |ΔCT|, AI) on 110 keV images were significantly lower than on 120 kVp-like and 50 keV images (*P* < 0.001). The application of MARs further significantly improved image quality at both energy levels (*P* < 0.005). Subjective scores [presented as median (IQR)] confirmed that the 110 keV + MARs series provided the best image quality, which was significantly superior to all other groups (*P* < 0.001).

**Conclusions:**

The high-energy VMI (110 keV) of the dual-energy CT can effectively reduce metal artifacts, and the combination of the 110 keV and MARs algorithm could further improve the image quality, which provides great assistance for the review and follow-up of children with CFC after metal plate correction.

## Introduction

Congenital funnel chest (CFC), the most common thoracic developmental deformity in children, is primarily characterized by sternal depression, resulting in one or more concave areas and an abnormal chest contour, including sternal retraction and thoracic asymmetry (1–3). This deformity can impair cardiopulmonary function, leading to symptoms such as dyspnea and cardiac compression. Thoracoscopic minimally invasive correction is a widely used surgical intervention for CFC. Postoperative computed tomography (CT) is routinely performed to assess the position of the implanted plate and its relationship with adjacent tissues (4, 5).

The orthopedic steel plate, which spans a large area of the anterior chest wall, frequently induces severe metal artifacts on conventional CT scans, significantly compromising diagnostic image quality (6, 7). Dual-energy CT (DECT) offers a solution through its monoenergetic (VMI) imaging and metal artifact reduction (MAR) algorithms (8, 9). Although DECT allows for VMI reconstruction across a wide energy range (40–140 keV), the optimal reconstruction setting for pediatric CFC patients following plate correction remains undetermined (10, 11).

Metal artifacts in conventional CT, primarily caused by beam hardening, photon starvation, and scatter, manifest as streaks and dark bands that obscure anatomical details and compromise quantitative accuracy, thereby posing a significant challenge to postoperative assessment (6). In response, various Metal Artifact Reduction (MAR) algorithms have been integrated into clinical CT systems (7). These algorithms typically function during image reconstruction or as post-processing steps by identifying and correcting corrupted projection data or interpolating missing data around metallic objects. Although beneficial in many cases, traditional MAR techniques can introduce secondary artifacts, blur tissue boundaries, or prove inadequate for very large or complex implants (8).

Spectral CT provides an alternative strategy through virtual monoenergetic imaging (VMI) (9). This technique decomposes the polychromatic x-ray spectrum into material-specific information, thereby enabling the reconstruction of images that simulate attenuation at a single, selectable energy level (keV). High-keV VMI is particularly effective at reducing beam-hardening—a primary source of metal artifacts—as higher-energy photons are less susceptible to differential absorption (10). Moreover, combining high-keV VMI with dedicated MAR algorithms has demonstrated a synergistic effect, offering superior artifact suppression in various clinical scenarios involving metallic implants, including dental work, orthopedic prostheses, and coils (11).

However, the optimal application of these techniques for postoperative imaging in pediatric CFC patients with stainless steel plates remains underexplored. Specifically, the most effective monoenergetic energy level and the added value of applying MARs at that level have not been established. Factors such as plate material, dimensions, location, and the smaller pediatric body habitus may significantly influence artifact characteristics and the efficacy of reduction strategies. Consequently, there is a clear need to develop evidence-based imaging protocols tailored to this specific population and implant type to ensure optimal diagnostic quality during postoperative follow-up.

This study aimed to compare the image quality of conventional mixed-energy imaging and dual-energy CT-based virtual monoenergetic imaging (VMI), evaluate the efficacy of VMI and a metal artifact reduction (MAR) algorithm, and determine the optimal VMI reconstruction level for pediatric CFC patients following metal plate correction.

## Materials and methods

### Patients information

We retrospectively analyzed children with CFC who underwent thoracoscopic minimally invasive correction at Xuzhou Children's Hospital between January 2022 and August 2023. The inclusion criteria were: (1) age 4–17 years; (2) history of thoracoscopic minimally invasive funnel chest correction; and (3) implantation of a specific stainless steel plate (Biomet Microfixation, model 01-3170). Patients were excluded for any of the following: (1) tracheotomy, airway stenosis, airway compression, or pneumothorax; (2) cyanotic congenital heart disease; (3) central respiratory failure, severe muscle weakness, or absence of voluntary diaphragmatic activity; or (4) terminal disease or death.

Ultimately, a total of 114 children with CFC were enrolled in the study. The cohort consisted of 99 males and 15 females, with an age range of 6–17 years and a mean age of 10.89 ± 3.99 years. The implanted funnel chest correction steel plate was made of stainless steel and measured 25.4 cm in length and 1.27 cm in width.

This study received full approval from the Institutional Review Board of Xuzhou Children's Hospital (Approval Number: 2022-05-14-H14) with a waiver of informed consent for this retrospective analysis. The research was conducted in accordance with local regulations, institutional policies, and the ethical principles of the Declaration of Helsinki (12). The analysis utilized exclusively anonymized, non-contrast chest CT scans obtained as part of standard postoperative follow-up for funnel chest correction. No additional procedures were performed for research purposes. The IRB determined the study posed minimal risk. All directly identifiable information was permanently removed from the research data prior to analysis, and datasets were stored on secure, encrypted servers with access restricted to authorized investigators.

### CT scan methods

All chest scans were performed on a 256-row CT scanner (Revolution 256 CT; GE Healthcare, Milwaukee, WI, USA). The scanning parameters were as follows: tube voltage with instantaneous switching between 80 and 140 kVp (switching time, 0.25 ms), tube current of 200 mA, slice thickness of 5 mm, and a display field of view (DFOV) of 25–40 cm. The reconstruction matrix was 512 × 512, with a rotation speed of 0.5 s/r, a pitch of 0.984, and adaptive statistical iterative reconstruction (ASIR-V) set at 50%.

### Radiation dose estimate

The radiation dose parameters for all CT examinations were recorded and analyzed. The volume computed tomography dose index (CTDIvol) and dose-length product (DLP) were automatically generated and archived by the CT system for each scan. The effective dose (ED) was estimated to provide a more patient-specific risk assessment. The ED was calculated using the formula: ED = DLP × k, where k is a age-specific conversion coefficient. Given that our cohort consisted of children, the k value of 0.039 mSv·mGy⁻^1^·cm⁻^1^ was applied, as recommended by the 2020 European Commission guidelines on diagnostic reference levels for pediatric imaging for chest CT in the 10-year-old age group.

### Imaging analysis

**Figure 1:**
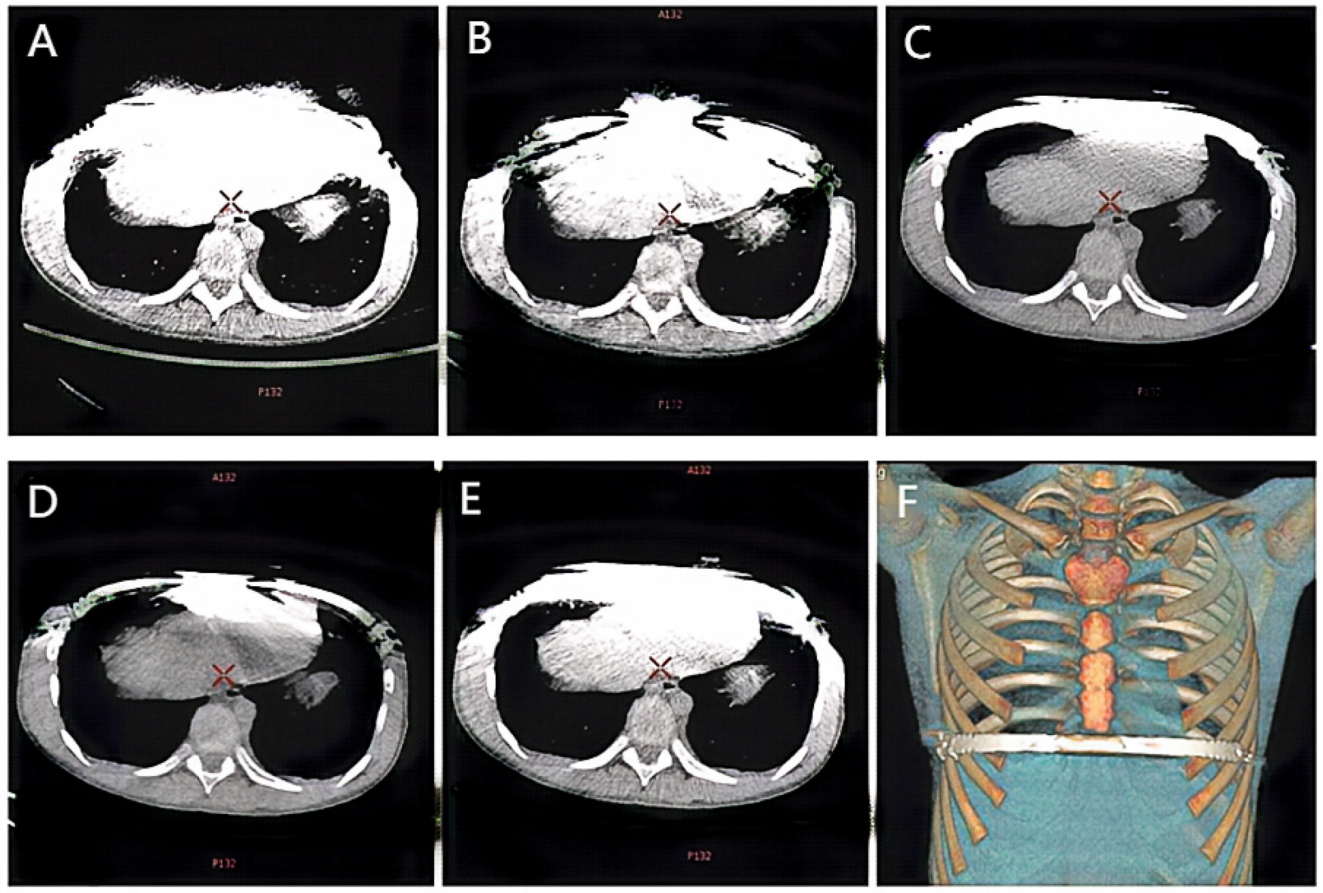
Ct images of the same child under different scanning conditions. **(A)** 50 keV; **(B)** 50 keV + MARs; **(C)** 110 keV; **(D)** 110 keV + MARs; **(E)** 120 kVp-like; **(F)** Volume reconstruction (VR) image reconstructed from 120 kVp like image.

The scans were performed using the Gemstone Spectral Imaging (GSI) mode, which is characterized by rapid kV switching between 80 kVp and 140 kVp during a single scan acquisition. This provides the necessary paired low-energy and high-energy projection data. All images were reconstructed on an AW 4.6 workstation (GE Healthcare, Milwaukee, WI, USA). The raw spectral projection data were used to reconstruct virtual monoenergetic images (VMI) at 50 keV and 110 keV, a mixed-energy image simulating a conventional 120-kVp scan (120-kVp-like), and all series were reconstructed with a 1.25 mm slice thickness. This reconstruction process involves material decomposition (into water and iodine basis material pairs) followed by the synthesis of images representing the calculated linear attenuation at the desired monochromatic energy (keV). Subsequently, metal artifact reduction (MARs) processing was applied to generate the 50 keV + MARs and 110 keV + MARs image series. Figure 1 illustrates examples of the five reconstructed image series: (A) 50 keV, (B) 50 keV + MARs, (C) 110 keV, (D) 110 keV + MARs, and (E) 120 kVp-like.

CT and standard deviation (SD) values were measured on 1.25-mm-thick images displayed at a mediastinal window setting (width, 400 HU; level, 40 HU). Circular regions of interest (ROIs) with an area of 60–80 mm^2^ were placed in three locations: the artifactual dark area at one end of the plate (recorded as CT_1_ and SD_1_), the adjacent chest wall muscles (CT_2_, SD_2_), and the erector spinae muscle (CT_3_, SD_3_). Each measurement was repeated three times and averaged. The absolute difference between CT_2_ and CT_3_ (|ΔCT| = |CT_2_ - CT_3_|) and the artifact index (AI = [SD_2_^2^ - SD_3_^2^]^(1/2)^) were then calculated.

The CT and standard deviation (SD) values were measured on the images of the mediastinal window (window width 400 HU, window level 40 HU) by a single senior radiologist with over 10 years of experience in pediatric imaging. To ensure perfect consistency and comparability across the five different image series, the circular regions of interest (ROIs) (area 60–80 mm^2^) were first meticulously placed on the three predefined locations on the 120 kVp-like images. The precise coordinates and dimensions of these ROIs were then copied and pasted onto the exact same anatomical locations on the other four image series (50 keV, 50 keV + MARs, 110 keV, 110 keV + MARs) using the “Copy and Paste” function on the AW 4.6 workstation. This automated process guaranteed that all ROIs were identical in position and size. The measurements were repeated three times for each location, and the average value was calculated and recorded as CT_1_, CT_2_, CT_3_, and SD_1_, SD_2_, and SD_3_, respectively. The absolute value of the difference between CT_2_ and CT_3_ (|ΔCT| = |CT_2_ - CT_3_|) and the artifact index (AI) [AI = (SD_2_^2^ - SD_3_^2^)^(1/2)^] were then calculated.

### Subjective score

All five image series were independently read and scored by two senior radiologists, and the image score criteria are as follows (13): (1) Score 1 is defined by numerous artifacts near and distant from the implant, poorly displayed metal edges, and poorly demarcated adjacent tissues, rendering the image non-diagnostic; (2) Score 2 is defined by extensive periprosthetic artifacts and few distant artifacts, with poorly defined metal edges and indistinguishable adjacent tissues, rendering the image non-diagnostic; (3) Score 3 is defined by moderate periprosthetic artifacts, blurring of the metal shape/edges and tissue demarcation, yet the image is considered diagnostically usable; (4) Score 4 is defined by mild artifacts and slightly blurred metal margins, with distinguishable tissue demarcation, making the image suitable for diagnosis; (5) Score 5 is defined by no or minimal artifacts, clear metal edges with regular morphology, and sharp tissue demarcation, providing excellent diagnostic quality. For analysis, a score of 4 or higher was considered diagnostically acceptable. In cases of initial disagreement between the two radiologists (defined as a score difference greater than one point), a consensus reading was held. During this session, the radiologists reviewed the images together, discussed their assessments with reference to the scoring criteria, and agreed upon a final consensus score for statistical analysis.

### Statistical analysis

Statistical analysis was performed using SPSS software (version 26.0; IBM Corp., Armonk, NY, USA). The normality of distribution for all continuous variables was assessed using the Shapiro–Wilk test. Since the data from all five image groups violated the normality assumption (*P* < 0.05), continuous data are expressed as median and interquartile range (IQR).

For comparisons of objective metrics (CT numbers, SD, |ΔCT|, and AI) across the five matched image series (which are related measures), the non-parametric Friedman test was first applied as the omnibus test. If a significant difference was indicated by the Friedman test, *post hoc* pairwise comparisons were conducted using the Wilcoxon signed-rank test. A Bonferroni correction for multiple comparisons was applied, resulting in an adjusted significance level of *P* < 0.005.

The inter-reader agreement for the subjective image quality scores between the two radiologists was evaluated using the Weighted Kappa (κ) statistic. The strength of agreement was interpreted as follows: *κ* ≤ 0.20, slight; 0.21–0.40, fair; 0.41–0.60, moderate; 0.61–0.80, substantial; and 0.81–1.00, almost perfect agreement.

The normality of the subjective scores was assessed using the Shapiro–Wilk test, which confirmed a non-normal distribution for all data sets (*P* < 0.001). Therefore, comparisons of subjective scores across the five image groups were performed using the non-parametric Friedman test. If a significant difference was found, *post hoc* pairwise comparisons were conducted using the Wilcoxon signed-rank test, with a Bonferroni correction applied.

## Results

### Comparison between virtual monoenergetic images and conventional 120 kVp-like images

#### Low-energy (50 keV) VMI vs. 120 kVp-like images

The objective metrics of the 50 keV images demonstrated a significantly higher artifact burden compared to the conventional 120 kVp-like images. The CT values, SD values, |ΔCT| and AI values of chest wall muscles and erector spinae muscles on 50 keV images were significantly higher than those on the 120 kVp-like images (*P* < 0.05, refer to Tables 1, 2). Subjectively, the 50 keV images received the lowest scores from both radiologists due to severe artifacts and noise.

**Table 1:** CT1, CT2, CT3, |ΔCT| values for each group of images.

Image series	CT_1_	CT_2_	CT_3_	|△CT|
120 kVp-like	−809.73 (−1,020.16, −356.26)	120.44 (−8.60, 239.75)	83.74 (46.19, 144.25)	39.04 (0.08, 153.03)
50 keV	−1,017.3 (−1,024.00, −755.25)	257.55 (−133.68, 594.61)	142.22 (47.07, 310.62)	118.35 (6.87, 434.98)
50 keV + MARs	−874.42 (−1,024.00, −202.88)	108.42 (−56.68, 237.38)	108.34 (42.59, 204.86)	32.25 (1.32, 193.35)
110 keV	−298.60 (−792.87, −32.95)	62.08 (6.91, 104.86)	58.15 (39.76, 79.35)	12.95 (0.17, 54.52)
110 keV + MARs	−81.94 (−483.19, 77.77)	48.89 (−21.26, 90.14)	57.20 (34.94, 86.59)	10.31 (0.19, 80.76)

**Table 2:** Comparison of CT1, CT2, CT3, |ΔCT| values of images in each group.

Comparison	50 keV & 50 keV + MARs				110 keV & 110 keV + MARs			
	CT_1_	CT_2_	CT_3_	|△CT|	CT_1_	CT_2_	CT_3_	|△CT|
Z	−8.65	−8.94	−8.89	−8.14	−8.75	−6.61	−1.31	−0.54
*P*	< 0.001	<0.001	<0.001	<0.001	<0.001	<0.001	0.19	0.59
	120 kVp-like & 50 keV				120 kVp-like & 110 keV			
	CT_1_	CT_2_	CT_3_	|△CT|	CT_1_	CT_2_	CT_3_	|△CT|
Z	−9.27	−8.97	−9.26	−9.24	−9.27	−8.97	−9.26	−7.41
*P*	<0.001	<0.001	<0.001	<0.001	<0.001	<0.001	<0.001	<0.001

#### High-energy (110 keV) VMI vs. 120 kVp-like images

In contrast, the 110 keV images exhibited superior artifact reduction capabilities. The aforementioned objective metrics (SD, |ΔCT|, AI) for the 110 keV images were significantly lower than those on the 120 kVp-like images (*P* < 0.05, refer to Table 3). The subjective scores for the 110 keV series were markedly higher than those for both the 120 kVp-like and 50 keV images.

**Table 3:** Sd2, SD3, AI values for each group of images.

Image series	SD_2_	SD_3_	AI
120 kVp-like	31.68 (16.20, 115.74)	19.38 (10.23, 30.90)	25.37 (5.29, 113.58)
50 keV	62.63 (28.55, 408.93)	34.17 (17.72, 60.17)	51.70 (6.92, 406.97)
50 keV + MARs	42.38 (23.78, 111.04)	28.24 (16.61, 49.24)	28.77 (4.84, 109.31)
110 keV	20.01 (10.82, 78.66)	13.11 (6.99, 19.73)	13.94 (0.76, 78.24)
110 keV + MARs	15.80 (9.26, 73.62)	11.11 (6.71, 34.05)	10.36 (0.92, 73.23)

### Additional value of the MARs algorithm

#### Effect of MARs on low-energy VMI (50 keV + MARs vs. 50 keV)

The application of the MARs algorithm on the 50 keV images led to a significant reduction in artifacts. The SD, |ΔCT|, and AI values on the 50 keV + MARs images were substantially lower than those on the 50 keV images without MARs (*P* < 0.05, refer to Table 3). This objective improvement was also reflected in the subjective evaluation.

#### Effect of MARs on high-energy VMI (110 keV + MARs vs. 110 keV)

Similarly, the MARs algorithm provided a significant further improvement on the already favorable 110 keV images. The objective metrics on the 110 keV + MARs images were the lowest among all groups, showing a statistically significant reduction compared to the 110 keV images alone (*P* < 0.05, refer to Table 4). Consequently, the 110 keV + MARs image series received the highest subjective scores from both readers, indicating excellent image quality with minimal artifacts.

**Table 4:** Comparison of SD2, SD, AI values of images in each group.

Comparison	120 kVp-like & 50 keV			120 kVp-like & 110 keV		
	SD_2_	SD_3_	AI	SD_2_	SD_3_	AI
Z	−9.27	−9.27	−9.26	−9.27	−9.24	−9.21
*P*	<0.001	<0.001	<0.001	<0.001	<0.001	<0.001
	50 keV & 50 keV + MARs			110 keV & 110 keV + MARs		
	SD_2_	SD_3_	AI	SD_2_	SD_3_	AI
Z	−8.59	−6.63	−7.85	−5.85	−5.83	−4.05
*P*	<0.001	<0.001	<0.001	<0.001	<0.001	<0.001

### Subjective scores

The subjective scores for all image series were non-normally distributed (Shapiro–Wilk test, all *P* < 0.001) and are therefore presented as median (IQR). The scores differed significantly among the groups (Friedman test, *P* < 0.001). *Post hoc* analysis revealed that the 110 keV + MARs series [median (IQR): 5 (5-5)] received significantly higher scores than all other groups (Wilcoxon test, all *P* < 0.005 after Bonferroni correction) (refer to Table 5).

**Table 5:** Subjective scoring of the five image series assessed by two radiologists.

Image Series	Radiologist 1 [Median (IQR)]	Radiologist 2 [Median (IQR)]
120 kVp-like	3 [2–3]	3 [2–3]
50 keV	2 [1–2]	2 [2–2]
50 keV + MARs	3 [3–4]	3 [3–4]
110 keV	4 [4–5]	4 [4–5]
110 keV + MARs	5 [5–5]	5 [5–5]
Overall *P*-value	*P* < 0.001 (Friedman test)	

Data are presented as median [interquartile range]. The overall difference among the five image series was statistically significant (Friedman test, *P* < 0.001). *Post hoc* pairwise comparisons with Wilcoxon signed-rank test (Bonferroni-corrected) revealed that the subjective score of the 110 keV + MARs series was significantly higher than that of all other groups (all *P* < 0.005).

**Figure 2:**
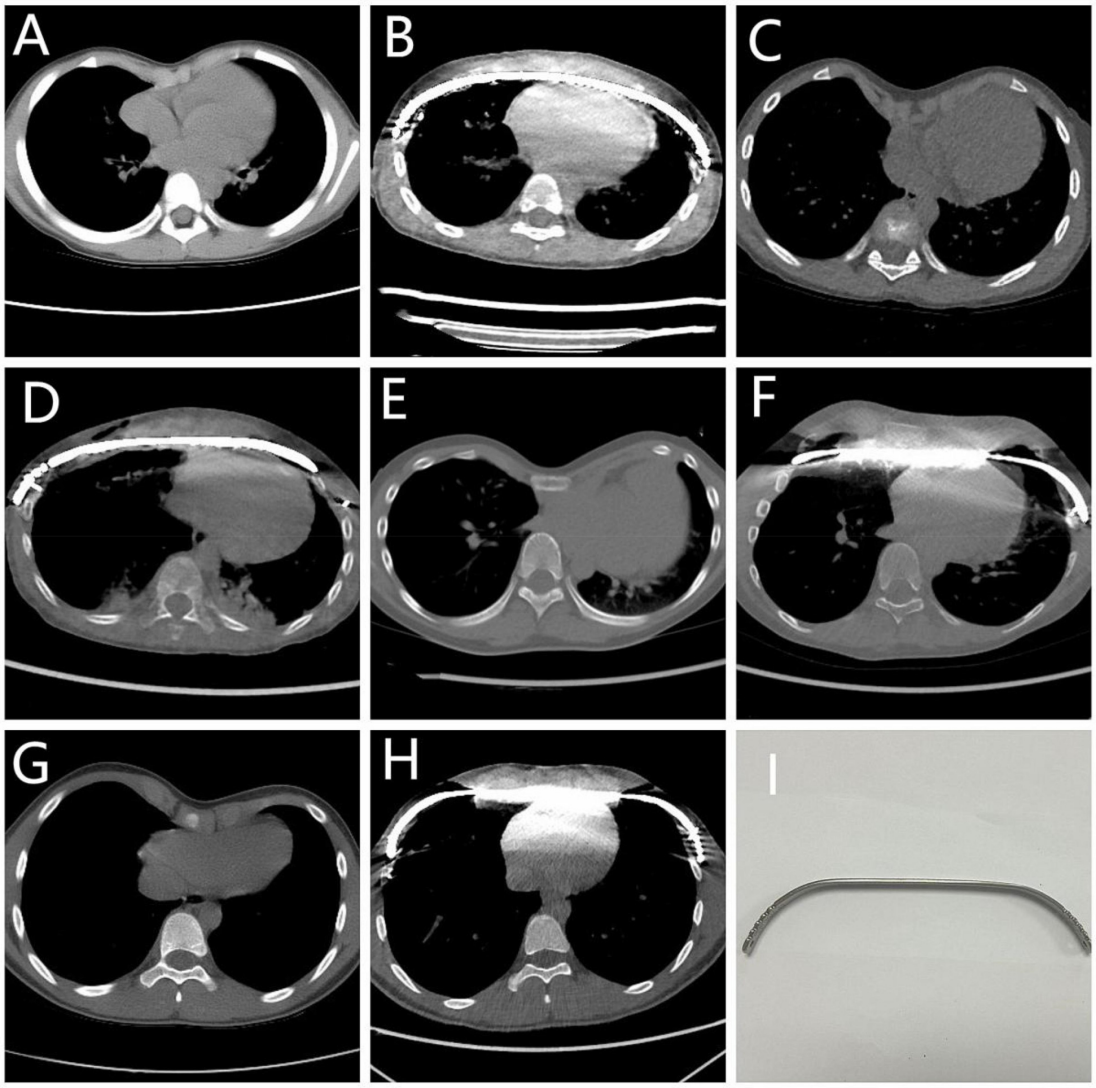
The CT images of four patients before and after thoracoscopic minimally invasive funnel chest correction surgery. **(A,B)** Male, 4 years old, preoperative and postoperative Haller index: 3.58, 2.99. **(C,D)** Male, 7 years old, preoperative and postoperative Haller index: 3.51, 2.45. **(E,F**) Male, 12 years old, preoperative and postoperative Haller index: 16.68, 3.48. **(G,H)** Male, 15 years old, preoperative and postoperative Haller index: 3.90, 2.63. **(I)** Orthopedic steel plate used for surgery, made of stainless steel. Annotation: Haller index is used for grading the severity of funnel chest, measured on CT by dividing the transverse diameter of the chest at the lowest point of the depression by the distance between the thoracic spine, and Haller index greater than 3.2 may require surgical treatment.

Pre- and postoperative CT images for four representative clinical cases are illustrated in Figure 2.

## Discussion

The primary findings of this study demonstrate that for pediatric patients with congenital funnel chest (CFC) following Nuss procedure with stainless steel plate implantation, high-energy virtual monoenergetic imaging (VMI) at 110 keV from spectral CT effectively reduces metal artifacts compared to conventional mixed-energy (120 kVp-like) imaging. Furthermore, the application of the metal artifact reduction (MARs) algorithm provides significant additional improvement in image quality at both 50 keV and 110 keV levels, with the combination of 110 keV and MARs yielding the best overall subjective and objective outcomes. This finding offers an optimized imaging protocol for postoperative assessment.

Dual-energy CT (DECT) is an advanced imaging technology enabling multi-parameter quantitative analysis, which has seen expanding clinical application in recent years (14–16). Its virtual monoenergetic imaging (VMI) technique leverages high- and low-energy datasets acquired during a single scan to reconstruct images simulating x-ray attenuation at a specific keV level. By providing superior material characterization, VMI yields richer diagnostic information and enhances diagnostic accuracy (17–19). The fundamental workflow begins with data collection, which involves acquiring paired high- and low-voltage (140 kVp and 80 kVp) projections. This data is then processed to generate linear absorption projection data, followed by material decomposition to produce material density projection data. The final steps involve reconstructing the base material map and subsequently synthesizing the virtual monoenergetic image at the desired energy level (20, 21).

Congenital funnel chest (CFC) is a common thoracic developmental anomaly in children (22, 23). Minimally invasive thoracoscopic correction is the current preferred surgical treatment. Postoperative monitoring of chest wall morphology and steel plate position requires regular CT follow-up (24, 25). However, conventional CT is inevitably compromised by metal artifacts (26). Virtual monoenergetic imaging (VMI) from dual-energy CT (DECT) has been developed specifically to mitigate such artifacts. According to the literature, the material, size, and placement position of metal implants are important factors affecting metal artifacts (27). Although the present study, by design, focused on a single implant type to control for these variables, the thickness and density of metals are known to be positively correlated with the severity of metal artifacts. The stainless steel plate used for the corrective surgery in this study measured 25.4 cm in length and 1.27 cm in width. On the axial images, the steel plate, which has a moderate physical thickness but a long length (25.4 cm), spans across a wide area of the anterior chest wall. Under conventional CT scanning, pronounced metal artifacts obscure the margins of the plate and adjacent soft tissues, compromising diagnostic assessment.

From a technical perspective, VMI synthesizes images at specific keV levels through material decomposition. It is established that higher keV photons penetrate metal more effectively, reducing artifacts caused by beam hardening, which is consistent with our observation that the 110 keV images exhibited a significantly lower artifact index (AI) than both conventional and low-energy (50 keV) images. Building upon this principle, our study further identifies the optimal imaging parameters for this specific implant and patient population.

All scans were performed in DECT spectral mode and reconstructed into 120 kVp-like, 50 keV, and 110 keV VMI series for comparing artifact reduction across energy levels. Additional 50 keV + MARs and 110 keV + MARs images were created to assess the algorithm's incremental value. Results confirmed high-energy VMI's superior artifact reduction over low-energy and conventional imaging (28). MARs is a post-processing technique that corrects metal-corrupted data via iterative algorithms to diminish artifacts (29). When combined with high-keV VMI [which reduces beam hardening (30)], they work synergistically to improve image quality and diagnostic accuracy. Our findings confirm that high-energy images best minimize artifacts and their effect on soft tissue CT values, with MARs providing further improvement.

Furthermore, it is imperative to state that the inclusion of the low-energy (50 keV) VMI series in our study was not to propose it as a viable clinical option for this population, but rather was driven by three key methodological considerations. First, the 50 keV images served as an internal “negative control”, explicitly delineating the worst-case scenario of maximum artifact burden. This provided an essential baseline for quantifying the magnitude of improvement offered by both high-energy VMI and the MARs algorithm. Second, demonstrating the efficacy of the MARs algorithm under the most challenging, high-artifact conditions of low-energy VMI constitutes a more rigorous test of its robustness and general utility, compared to solely showcasing its effect on an already-favorable high-energy image. Finally, by presenting the complete spectrum of image quality from low- to high-energy VMI, both with and without MARs, our study provides a more comprehensive and compelling evidence chain for the artifact-reducing capabilities of spectral CT.

Our findings align with the established principle that high-energy VMI reduces beam-hardening artifacts (7, 10, 11). For instance, studies in adults with hip prostheses have similarly shown the superiority of high-keV VMI combined with MARs (8, 11). However, our study uniquely focuses on a specific pediatric population with a distinct implant and a standardized surgical context. While Pessis et al. (7) demonstrated the general benefit of VMI for metal artifact reduction, they did not focus on pediatric thoracic applications. Similarly, Zhao et al. (8) optimized VMI for hip arthroplasty in adults, which involves different metal types, artifact geometry, and patient size. The smaller body habitus and different biomechanical environment in children may alter artifact characteristics, necessitating tailored protocols. Therefore, our work provides novel, population-specific evidence that extends the general principles of spectral CT to the postoperative assessment of CFC, delivering a concrete imaging protocol (110 keV + MARs) that can be directly adopted in clinical practice for these children.

This study has several limitations. First, its retrospective design carries inherent biases. Second, although DECT enables reconstruction of virtual monoenergetic images (VMI) across a continuous spectrum (40–140 keV), our analysis was restricted to 50 and 110 keV; future studies should include a broader range of energy levels. Third, the moderate inter-reader agreement in subjective scoring suggests that employing more readers or refining the scoring criteria could improve consistency in future investigations, although our primary findings are supported by objective metrics. Fourth, we evaluated only the vendor-provided MARs algorithm due to its clinical relevance; future work could explore advanced techniques like deep learning-based methods, provided they are clinically practical. Finally, this study was limited to a single type and size of stainless steel plate. While this homogeneity allowed for a controlled evaluation of imaging parameters, it limits the generalizability of our findings to other implant materials or dimensions. Future studies incorporating a variety of implants are needed to establish comprehensive, implant-specific imaging protocols.

## Conclusions

In summary, high-energy VMI (110 keV) from DECT effectively reduces metal artifacts, and the addition of the MARs algorithm further enhances image quality. This optimized protocol provides significant value for the postoperative assessment and follow-up of children with CFC, demonstrating broad clinical applicability.

## Data Availability

The raw data supporting the conclusions of this article will be made available by the authors, without undue reservation.
